# Triboelectric Mechanism of Oil‐Solid Interface Adopted for Self‐Powered Insulating Oil Condition Monitoring

**DOI:** 10.1002/advs.202207230

**Published:** 2023-02-24

**Authors:** Song Xiao, Haoying Wu, Nan Li, Xiangyu Tan, Haocheng Deng, Xiaoxing Zhang, Ju Tang, Yi Li

**Affiliations:** ^1^ School of Electrical Engineering and Automation Wuhan University Wuhan Hubei 430072 China; ^2^ State Grid Tianjin Electric Power Research Institute Tianjin 300392 China; ^3^ Electric Power Research Institute Yunnan Power Grid Co., Ltd. Kunming Yunnan 650217 China; ^4^ Hubei Engineering Research Center for Safety Monitoring of New Energy and Power Grid Equipment Hubei University of Technology Wuhan Hubei 430068 China

**Keywords:** electrical double layer, oil‐solid contact electrification, trace water detection, transformer oil, triboelectric nanogenerator

## Abstract

The liquid‐solid contact electrification mechanism has been explored in the aqueous solution system, but there are few systematic studies on oil‐solid triboelectrification. Herein, an oil droplet triboelectric nanogenerator (Oil‐droplet TENG) is designed as the probe to investigate the charge transfer properties at oil‐solid interface. The charge transfer kinetics process is disclosed by the electrical signals produced, showing that the electron species initially predominated the oil‐solid triboelectrification. The molecular structure and electronic properties of oil also affect triboelectric performance. Further, the charge transfer principle in multi‐component liquid mixture during the electric double layer (EDL) development process is proposed to explain the component competition effect. As a proof of concept, a tubular‐TENG is designed as a self‐powered sensor for transformer oil trace water detection. The device demonstrates high water sensitivity with a detection limit of 10 µL L^−1^ and a response range of 10–100 µL L^−1^. This work not only reveals the oil‐solid triboelectric and charge transfer competition mechanism in EDL, but also open up a new channel for real‐time online monitoring of trace water in transformer oil, which holds promise for information perception and intelligent operation of transformers in the power industry.

## Graphical abstract

The oil‐solid contact electrification charge transfer kinetics is investigated based on oil‐droplet triboelectric nanogenerator (TENG) and multi‐component electric double‐layer model is proposed. The role of molecular properties for charge transfer is discovered. A self‐powered trace water detection sensor (tubular‐TENG) is developed, bringing new pathways for transformer oil monitoring.

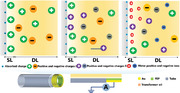

## 1 Introduction

Contact electrification (CE) or triboelectrification universally exists at different interfaces, including liquid‐solid (L‐S) materials.^[^ ^1^, ^2^, ^3^ ^]^ The charge transfer mechanism during L‐S contact electrification is of broad interest and importance. The invention and development of triboelectric nanogenerator (TENG) offer a fresh perspective on the origin of contact electrification.^[^ ^4^, ^5^ ^]^ TENG could convert kinetic energy from fluid droplets, flows, waves, etc. into electricity by combining contact electrification with electrostatic induction.^[^ ^6^, ^7^, ^8^ ^]^ Importantly, droplet‐TENG can be utilized as a probe to investigate the microscopic L‐S triboelectric mechanism, specifically the charge transfer at the liquid‐solid interface that determines the macroscopic output performance.^[^ ^9^, ^10^, ^11^ ^]^ Several droplet‐TENGs have been designed to reveal the cumulative process and carrier species (electrons or ions) of charge transfer.^[^ ^12^, ^13^, ^14^ ^]^ For instance, the water‐solid CE charge transfer process has been experimentally proven by Wang's group to involve both electrons and ions, with electron transfer predominating (providing 90% of total charges).^[^ ^15^, ^16^ ^]^ The ion and electron transfer are independent, and the former will generate “sticky” charges that cannot be removed from the surface.^[^ ^15^ ^]^ Further, the “Two‐step” electric double layer (EDL) formation process in an aqueous solution system has been proposed. The electron transfer occurs first owing to the electron cloud overlap of water and solid molecules, followed by the attraction of free ions due to the electrostatic interactions to form the EDL.^[^ ^4^, ^17^ ^]^ Besides, the ionization reactions such as H_2_O+ H_2_O^+^ → OH + H_3_O^+^ also exist during the EDL formation, which is crucial for the transpiration‐driven electrokinetic power generator.^[^ ^18^, ^19^ ^]^ The CE mechanism of the aqueous system has been thoroughly exposed by recent investigations, but additional investigation into other liquids like oil is still needed.

Oils are commonly recognized as the “lifeblood” of modern industry. Numerous oils, including insulating and lubricating oil, are widely used in the power industry, transportation, consumption, etc. For instance, lubricating oil is crucial for controlling wear and friction at moving mechanical contacts because it creates a fluid oil coating that increases machine longevity.^[^ ^20^, ^21^ ^]^ Insulating oil is employed as electrical insulation and heat dissipation medium for transformers adopted in the power industry, which usually works in a flowing state accompanied by the oil‐solid interface contact electrification.^[^ ^22^, ^23^ ^]^ For the transformer, the oil‐flow electrification endangers the insulation property of the oil‐paper insulation, which is a crucial harmful factor to equipment secure operation.^[^ ^24^ ^]^ Since oil and water have significant physiochemical differences in molecular structure, polarity, conductivity, and viscosity, the oil‐solid CE mechanism and charge transfer might be different.^[^ ^25^, ^26^ ^]^ Zhao et al. designed a liquid‐liquid TENG to explore the CE between transformer oil and water.^[^ ^27^ ^]^ They discovered that water molecules could capture electrons from oil and the electrons dominate charge transfer at the interface. To learn more about the oil‐solid CE process, particularly the charge‐transfer kinetics, further systematic investigations are needed.

Besides, oil is difficult to achieve absolute purity under long‐term operation. Intruding contaminants from thermal oxidation, dampness, wear debris, etc., usually exist in industrial oils.^[^ ^28^ ^]^ The impurities such as trace water change the oil composition, which will significantly impact the oil‐solid CE process, especially the EDL's charge transfer mechanism and development process. Thus, the identity of charge carriers and formation of EDL for oil‐containing impurities should be clarified. Further, the impurities in oil also provide helpful information for equipment operation conditions, similar to human blood for health assessment. To this end, the TENG‐based self‐powered sensor demonstrates a promising solution, and the pioneering studies were conducted by Shi's group. They realized lubricating oil condition monitoring by detecting the debris (carbon, metal), and water contaminants based on the oil‐solid TENG.^[^ ^29^ ^]^ The functional oleophobic coating further improved the sensitivity and durability.^[^ ^30^ ^]^ This finding opens a new path for oil‐solid energy harvesting and quality monitoring, but more research is needed to understand the microscopic mechanism.

In this study, we designed an oil‐droplet triboelectric nanogenerator (Oil‐droplet TENG) as the probe to characterize the CE properties at the oil‐solid interface. The electrical signals generated by different types of oils were obtained to reveal the dynamic charge transfer performance. The role of molecular electronic structure in triboelectrification was analyzed at the molecular level by density functional theory (DFT). Further, the charge transfer and EDL development principle of the multi‐component liquid mixture were proposed to explain the component competition effect. Finally, a Tubular‐TENG was fabricated as a self‐powered transformer oil trace water sensor, demonstrating high sensitivity with a detection limit of 10 µL L^−1^ and response range of 10–100 µL L^−1^. This study provides evidence for the electron as the dominant charge carrier for oil‐solid CE and the competition transfer mechanism in multi‐component EDL. It was also suggested and validated for the new route for real‐time online monitoring of trace water in transformer oil by Tubular‐TENG, promising for information perception and intelligent operation of oil‐immersed transformers in the power grid.

## 2 Results and Discussion

### 2.1 Oil‐Droplet TENG for Oil‐Solid CE Characterization

**Figure 1:**
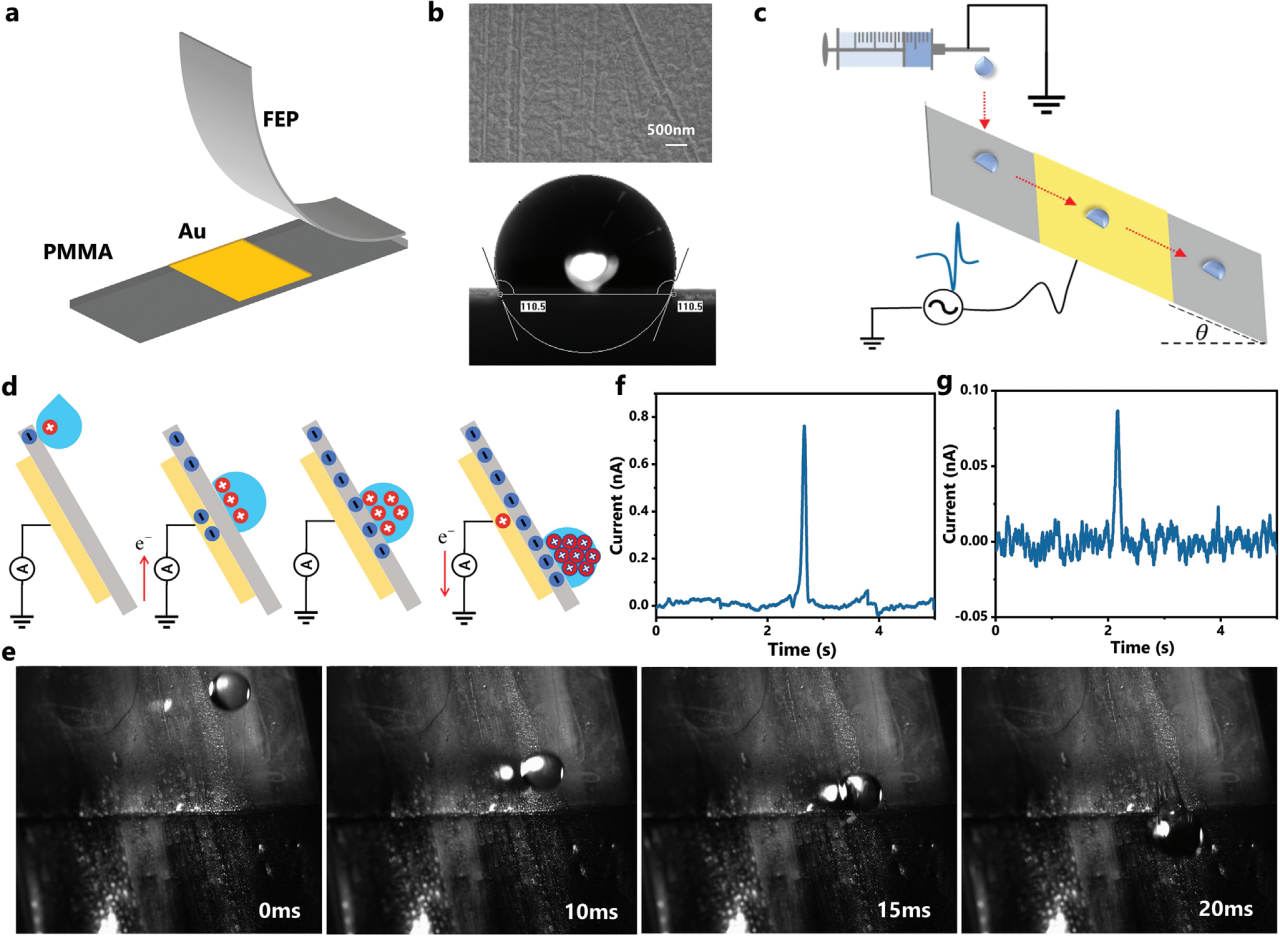
The fabrication and working mechanism of the Oil‐Droplet TENG. a) Structure of the Oil‐Droplet TENG. b) The surface morphology and oil contact angle (25^#^ transformer oil) of the oleophobic FEP film. c) The working mechanism of the Oil‐Droplet TENG. d) The charge transfer process of the Oil‐Droplet TENG. e) Snapshots of the oil droplet sliding on the oleophobic FEP film (25^#^ transformer oil). f,g) The output current waveform of the first and 40th oil droplets (25^#^ transformer oil).

The Oil‐Droplet TENG possessed a sandwich‐like structure that consisted of three layers, as shown in **Figure** **1**a. The top layer was the oleophobic‐treated fluorinated ethylene propylene (FEP) film for contact electrification with oil droplets. The middle layer was sputtered gold as the electrode for electrostatic induction, and the bottom layer was a polymethylmethacrylate (PMMA) plate as the substrate. The gold film covered the middle part of the oleophobic FEP film, and the signal induced on it was generated by oil sliding instead of falling. FEP was selected as a triboelectric active layer owing to its strong electron‐withdrawing ability and high stability, while its oleophilic characteristic enables oil droplet infiltration and discontinuities. Thus, the 1H,1H,2H,2H‐Perfluorodecyltriethoxysilane (POTS) was spray coated to obtain an oleophobic interface. POTS is a fluorinated alkyl silane that is frequently used to increase the substrate's wettability by reducing surface energy, which makes it easier to coat surfaces with hydrophobic or oleophobic properties by enriching them with ‐CF_3_ groups.^[^ ^31^, ^32^ ^]^ The surface morphology and oil contact angle of oleophobic FEP are shown in Figure 1b. The oil contact angle improved from 69.5° (Figure S1, Supporting Information) to 110.5° (25^#^ transformer oil), and the scanning electron microscopy (SEM)‐energy dispersive X‐ray spectroscopy (EDS) given in Figure S2, Supporting Information, verified the successful introduction of wealthy fluorine groups. The oil droplets can slide smoothly on the oleophobic FEP film, as shown in Movie S1, Supporting Information.

In this study, oil droplets (≈30 µL per drop, 30 drops per minute) were extruded from a syringe propelled by a microflow pump, falling freely from a grounded stainless‐steel needle (2 mm diameter) at a fixed height (≈0.8 cm from the film). The oleophobic FEP film below the needle keeps a large tilted angle of 80° to ensure the smooth sliding of oil droplets. The drop point of oil droplets on the oleophobic FEP film is about 1.5 cm away from the gold‐plated area, effectively isolating the induced electrical signal generated by oil droplets hit on the film, as shown in Figure 1c and Movie S2, Supporting Information. We further characterized the stability of POTS coating by conducting long‐term oil‐solid CE tests (6 h). The surface morphology barely changed, and the oil contact angle decreased from 110.5° to 109.3° owing to the residual oil (25^#^ transformer oil), which remains oleophobic (Figure S3, Supporting Information). In contrast, the oil contact angle of untreated commercial FEP film decreased rapidly from 69.5° to 8.2° after 120 s CE tests and the output signal was wholly submerged in the interference (Figures S4 and S5, Supporting Information). Thus, the oleophobic treatment is necessary to ensure a stable oil‐solid CE process.

Figure 1d shows the charge transfer process of the Oil‐Droplet TENG. As the oil droplet contacts with treated FFP, charges move from oil to FEP due to its strong electron‐withdrawing ability. Thus, the oil droplet and FEP film are positively and negatively charged, respectively. When it slides across the upper side of the gold‐plated area, the excess charge on the oil droplet leads to an induced charge and generates a positive pulse waveform output. Similarly, an opposite charge flow will be induced on the electrode when the oil droplet leaves the gold‐plated area. That is, pulse triboelectric signals with opposite polarity will be generated as they contact and leave the gold‐plated area's upper and bottom sides.

The oil droplets that have a low surface tension and a high viscosity might rupture and gradually turn into a fluid during the impact and bounce with oleophobic FEP film. Herein, the snapshot of oil droplets flowing through the oleophobic FEP film was captured by a high‐speed camera (15 000 frames per second), as shown in Figure 1e. When the oil droplet touched the oleophobic FEP at t = 2 ms, the drop partially spread to an ellipsoid structure (t = 10 ms) and then started to flow upon the surface (t = 15 ms). Upon sliding, the oil droplet head removed faster than the tail, demonstrating a trailing phenomenon and a little stain remained on the oleophobic FEP surface (t = 20 ms). Therefore, the output current signal of Oil‐Droplet TENG showed obvious positive pulse signals when it slid through the upper side of the gold‐plated area, while the negative pulse waveform was not obvious. Figure 1f,g shows the induced current signals generated by the first and 40th oil droplets. The positive peak value of the first drop current was 0.71 nA, which is significantly higher than the interference signal (≈ 0.01 nA). The positive peak value of the 40th drop current decreased to 0.09 nA, and the negative peak value was submerged in the interference signal. It is noteworthy that the signal induced on oleophobic FEP film is generated by oil sliding instead of falling. As demonstrated in Figure S6, Supporting Information, the first and 40th oil droplets created induced current signals that were substantially greater than the sliding signals, reaching 2.08 and 1.65 nA, respectively. This is explained by the fact that the falling‐induced signal consists of both the triboelectrification produced by oil sliding across the FEP layer and the kinetic energy‐induced impact/contact triboelectrification caused by oil droplets. The latter involves the conversion of kinetic energy impulse, bringing higher triboelectric output performance. Herein, we mainly focus on the CE process during oil‐solid sliding triboelectrification and primarily use the positive peak value change rule of the current signal to illustrate the principle of oil‐solid CE.

### 2.2 Oil‐Solid CE Development Mechanism

**Figure 2:**
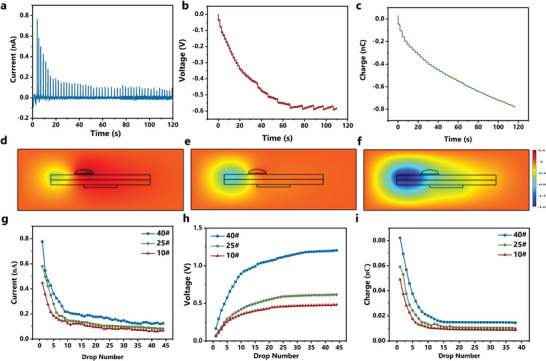
CE development process of oil droplets sliding across the FEP interface. a–c) The triboelectric output of (a) Short‐circuit current, (b) Open‐circuit voltage, and (c) Accumulated charge of 25^#^ transformer oil droplets. d–f): The potential distribution of oil droplets flowing through the FEP film under different conditions. (d) The first droplet, (e) The 5th droplet, (f) The 20th droplet. g–i): The triboelectric output of three types of transformer oils flow over the FEP interface. (g) Short‐circuit current, (h) Open‐circuit voltage, (i) Transferred charge.

The triboelectric performance of 25^#^ transformer oil and FEP polymer was examined in order to better understand the oil‐solid interface CE mechanism. The Oil‐Droplet TENG is a current source and the measured output current waveform is shown in **Figure** **2**a. The current peak value decreased from 0.71 to 0.09 nA with the increase in droplet number, indicating an inhibition effect on the oleophobic FEP surface during oil‐solid CE. The output voltage and transfer charge waveforms confirmed that this inhibition originated from CE surface charge accumulation. The detected discrete current signal reflected the electrostatic induction process, which belongs to the dynamic, repeatable process and each peak represented a corresponding droplet. As shown in Figure 2b, the open‐circuit voltage presented a stepwise rising trend from 0 to ≈ 0.6 V, demonstrating a negative saturated exponential rising trend with the oil droplet number. The open‐circuit voltage reflected the surface potential generated by the charge on the surface, whose value is proportional to the surface charge. Therefore, accumulating residual charge on the oleophobic FEP surface accounts for the inhibition effect. Figure 2c shows the measured charge as the meter is directly connected to the electrode of the Oil‐Droplet TENG, which belonged to the cumulative external charge by the oil droplet sliding over the surface. The waveform demonstrated a ladder‐like shape, and the peak difference of the adjacent step was the net transferred charge during sliding. Considering the trailing phenomenon of oil droplets, the CE charge transfer mainly occurred when it contacted the electrode area and the charge value during separation was submerged in the interference signal. It is worth noting that the measured charge contains the oil‐solid CE transferred charge and the electrostatic inducted charge. The CE transferred charge gradually decreased as the surface accumulated charge reached saturation, and the electrostatic induction part dominated. This process is similar to water‐solid CE, as reported by Wang et al.^[^ ^31^ ^]^

We hypothesize that oil‐solid triboelectrification follows a similar process as water‐solid CE and that both electrons, ions serve as charge carriers.^[^ ^32^, ^33^, ^34^ ^]^ The triboelectric active layer (oleophobic FEP film) was treated with an ion‐air gun and the insulating oil droplet was grounded to maintain its zero‐surface potential and electrically neutral. When the oil droplets slid over the polymer surface, the overlap of the electron clouds of oil and FEP molecules led to electron transfer first. That is to say, the oleophobic FEP film with strong electron capturing ability obtained electrons from oil droplets and ions were generated simultaneously. This process was recognized as the ionization reaction. At this stage, the electrons and ions attached to the solid interface work parallelly, changing the surface potential and forming the EDL. Specifically, ions with larger mass usually underwent chemical adsorption on the solid interface owing to the strong binding force, while electrons were less bounded. It is reported that ions adsorbed on the surface will form “sticky charges” that are hard to remove.^[^ ^15^ ^]^ Thus, surface charge and potential accumulation occurred as more oil droplets slid through the oleophobic FEP film. Meanwhile, ion adsorption on the solid surface would produce a “screen effect”, hindering the electron transfer process and reducing the total amount of transferred charge.^[^ ^16^ ^]^ Specifically, as the available charge positions on the solid surface are limited and the “sticky charges” permanently occupy the available charge positions, the number of transferred electrons would be inhibited. Therefore, the triboelectric output remained relatively stable as the charge accumulation reached saturation to the short‐circuit current. At this stage, electrons acted as dominant carriers for electrostatic induction.

The in‐detail charge transfer process of oil‐solid CE is shown in Figure S7, Supporting Information. When the first oil droplet flew through the uncharged oleophobic FEP film, the positive charge accumulated by the oil drop was the largest because there was no charge on the film to hinder the charge transfer. Meanwhile, the surface charge of oleophobic FEP film was the smallest, and the charge difference on the surface was the largest during the contact process. Therefore, the maximum current was generated to balance the electric neutrality when the oil droplet slid through the electrode area. Meanwhile, a reverse current pulse was generated to rebuild the electrically neutral when the oil droplet slid out of the electrode area. Some oil ions and electrons were adsorbed and the surface potential was built on the oleophobic FEP surface. As the number of oil droplets increased gradually, the residual charge on the surface accumulated and produced a screen effect, which would hinder the electron transfer process and reduce the total amount of transferred charge. Although the oil‐solid CE charge transfer process was continued, fewer ground electrons were needed for electric neutrality and the output current decreased, as shown in Figure S7b, Supporting Information. When the accumulated charge reached saturation (Figure S7c, Supporting Information), oil droplets sliding through the interface induced little ionization reaction and CE charge transfer. The electrostatically generated electrons dominated and the output current waveform reached a stable state at this stage.

Based on the charge distribution law proposed above, we further calculated the electric potential distribution of the triboelectric active layer considering the ion accumulation process based on COMSOL Multiphysics and compared it with the experimental results to verify the theoretical hypothesis. Three droplets at different periods were considered: 1) The first oil droplet slid past the upper side of the electrode area, which corresponded to the initial stage without ionization reaction (Figure 2d). 2) The 5th oil droplet slid past the upper side of the electrode area where the charge accumulation was unsaturated (Figure 2e). 3). The 20th oil droplet) slid past the upper side of the electrode, which belongs to the saturation stage (Figure 2f). As shown in Figure 2d, the oil droplet demonstrated the highest external potential when no ion was accumulated on the film, which hindered the charge transfer and generated the largest unbalance charge. Similarly, the external electric potential is the lowest when the ionization reaction or ion accumulation reaches saturation. A small charge mainly causes this in oil droplets as the surface is charge balanced. The above results were consistent with the experimental results, validating the oil‐solid CE development mechanism.

Generally, industry oil is fractionated product of petroleum, which mainly includes the paraffin base oil (mainly containing alkanes), the intermediate base oil, and the naphthene base oil. The insulating oil used in the power industry is divided into three categories (listed as 10^#^, 25^#,^ and 45^#^) according to the freezing point, in which the 10^#^, 45^#^ transformer oil is used in high temperature and cold areas, respectively and the 25^#^ transformer oil is the most used. They are essentially the combination of three types of hydrocarbon compounds in different content. The viscosity and oil contact angle of the above‐mentioned transformer oil is similar at 20–100 °C, as shown in Figures S8 and S9, Supporting Information. The triboelectric output performance of 10^#^, 25^#,^ and 45^#^ insulating oils were also explored. The short‐circuit current, voltage, and transferred charge met the order of 10^#^<25^#^<45^#^, indicating that the oil component types and the content will directly affect the oil‐solid triboelectric output performance (Figure 2g,h). It also inspired us to use triboelectric properties differences for oil component identification, which will be investigated in the following section.

### 2.3 Influence of Oil Composition on CE Process

**Figure 3:**
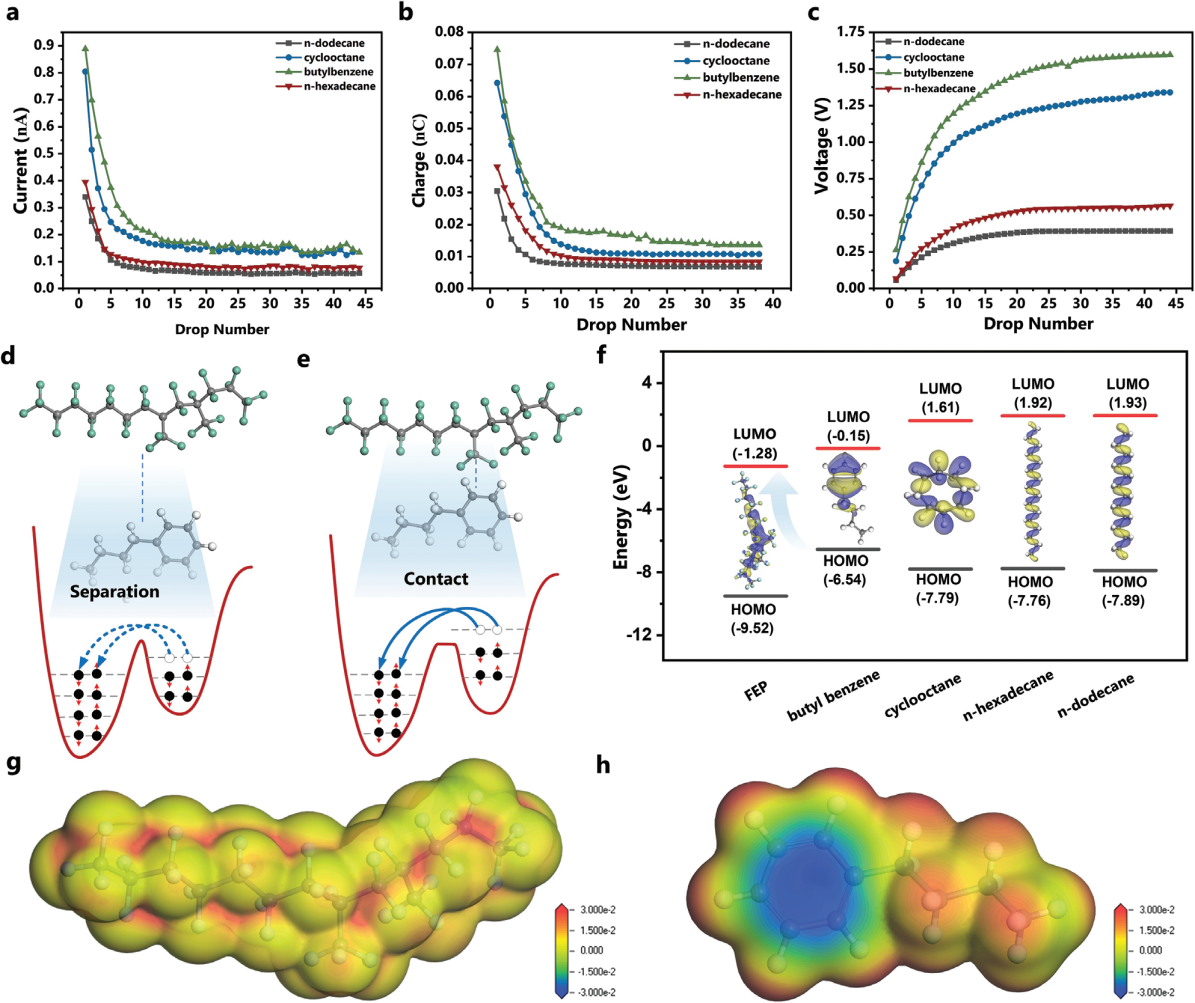
The influence of oil composition on CE properties. a–c): The triboelectric output of butylbenzene, cyclooctane, n‐hexadecane, and n‐dodecane. (a) Short‐circuit current, (b) Transferred charge, (c) Open‐circuit voltage. d,e): Electron cloud potential well model for oil‐solid CE process, demonstrating the molecular orbital (d) before and (e) during contact. f) The frontier molecular orbital (HOMO, LUMO) energy of the FEP and oil molecules. g) The molecular electrostatic potential distribution of FEP. h) The molecular electrostatic potential distribution of butyl benzene.

Typically, the composition of transformer oil mainly includes alkanes, cycloalkanes, and aromatic hydrocarbons. The oil‐solid CE process is essentially determined by the microscopic properties such as molecular structure, functional group, electronic properties, etc. To demonstrate the impact of oil composition on the CE process, we further evaluated the triboelectric performance of typical transformer oil components in this work. Specifically, we selected n‐dodecane on behalf of alkanes and compared it with n‐hexadecane to show the influence of carbon chain length. The cyclooctane, butyl benzene was considered as typical cycloalkanes, aromatic hydrocarbons. The oil contact angle of butylbenzene reaches 110.7°, followed by cyclooctane (110.4°), n‐hexadecane (108.4°), and n‐dodecanoic (101.2°) (Figure S10, Supporting Information). **Figure** **3**a–c displays the triboelectric output performance of several components. All the oil compositions demonstrated a similar output‐changing trend with transformer oils. The short‐circuit current, voltage increment, and net charge gradually decayed with the increase of oil droplet number, and the saturation value was achieved at the 10–15^th^ droplet. However, different components demonstrated unequal output at the saturation state. The following order applies to the saturation output characteristics of voltage, current, and charge: butyl benzene > cyclooctane > n‐hexadecane > n‐dodecanoic. In other words, aromatic hydrocarbon, cycloparaffin performs better CE performance than alkanes. The longer carbon chain also helped to increase output. This result was consistent with the triboelectric order of three types of transformer oils. Particularly, the output is lowest for the 10^#^ transformer oil since it primarily comprises alkanes. Cycloalkanes make up the majority of the 45^#^ transformer oil, which has the highest triboelectric output. The output and composition of 25^#^ transformer oil made from intermediate base oil range from 10^#^ to 45^#^.

According to the “electron‐cloud potential well” model proposed by Wang et al., the collision of liquid and solid molecules driven by pressure leads to the overlapping of electron clouds and cancels out the potential barrier between two molecules, which benefits electron transfer between molecules during the CE process.^[^ ^34^, ^35^ ^]^ As illustrated in Figure 3d, the large molecular distance between oil and FEP before contact built a strong barrier between them, which hindered the intermolecular charge transfer. Oil‐solid interfaces initial single potential well evolved into asymmetric double‐well potential as oil droplets moved across the oleophobic FEP sheet, allowing electron transfer between them (Figure 3e). The potential barrier gap determined the charge transfer intensity as well as the triboelectric output. From the point of molecular orbital, when they come into contact with each other, the electron acceptor provides empty orbitals and the electron donor donates the outermost electrons for charge transfer to reach a new equilibrium. After separation, electrons transferred to FEP will be kept owing to the rebuilt energy barrier, so that it is negatively charged after CE.

Generally, the lowest unoccupied molecular orbital (LUMO) and highest occupied molecular orbital (HOMO) are essential indicators for electron attractions or interactions. Herein, we calculated the HOMO, and LUMO of oil and FEP molecules based on the DFT to illustrate their charge transfer process. As shown in Figure 3f and Figure S11, Supporting Information, the LUMO of FEP is located at −1.28 eV with the electron orbital wave function widely distributed along the carbon chain, providing sufficient empty orbital sites to receive electrons. The HOMO energy level of butylbenzene, cyclooctane, n‐hexadecane, and n‐dodecane is −6.54, −7.79, −7.76, and −7.89 eV, respectively. The LUMO‐HOMO energy barrier between FEP and cyclooctane is 5.26 eV, which is the lowest compared to the other oil‐molecular pairs. The electron‐cloud potential well model suggests that when two atoms are separated by less than the equilibrium bonding distance, their electron clouds will overlap and repel one another. Additionally, because the two surfaces are in close contact, the electron clouds of the two atoms will overlap and lower the potential barrier.^[^ ^36^, ^37^ ^]^ The electron clouds overlap when the oil droplets move across the FEP film and come into touch with the FEP molecules. The energy used to migrate electrons from lower to higher orbitals was first reduced by the lowering of the barrier due to the overlap of the electron clouds, and the remaining energy came from the repulsive force generated when the interatomic distance was less than the equilibrium distance. Both energies were essentially derived from the kinetic energy of the oil droplets. Overall, the electron transfer between FEP and cyclooctane requires the lowest energy barrier, which coincides nicely with the CE test results. Besides, the HOMO electron orbital wave function of butylbenzene is concentrated around the benzene ring, suggesting the main occurrence site of oil‐solid interaction as well as electron transfer. The electron orbital wave function distribution of cyclooctane is more concentrated than the n‐hexadecane with chain structure (Figure S11, Supporting Information), which benefits the HOMO‐LUMO interaction and brings higher triboelectric output even though both have similar HOMO energy levels around 7.79 eV.

The electrostatic potential (ESP) provides an effective way to identify the high or low electron density regions in molecules, which can be used to explain and predict intermolecular electrostatic interactions. We also calculated the ESP distribution of FEP and various oil molecules. According to Figure 3g,h, the FEP molecule exhibits relative positive potential and the highest area locates around the carbon atoms in the chain, indicating its strong electron capture capability and electrostatic interaction with negative particles. The benzene ring in butylbenzene demonstrates the largest negative potential, which is prone to undergo electrostatic interaction with FEP positive area for charge transfer. However, the ESP distribution of cyclooctane, n‐hexadecane, and n‐dodecane exhibit little concentrated negative potential region compared to butylbenzene (Figure S12, Supporting Information), contributing to lower CE output.

Overall, we can conclude that the oil composition with higher HOMO energy level, more concentrated electron orbital wave function, and pronounced negative ESP distribution benefits the oil‐solid CE performance. The overlapped electron‐cloud model is an effective method for electron transfer mechanism explanation in oil‐solid CE.

### 2.4 Multi‐Components Competition Process in EDL

The EDL model is commonly utilized to illustrate the charge and potential distribution at the liquid‐solid interface, which is the other core problem in CE. Conventional EDL suggested that the charge transfer at the interface is owing to ions adsorption or electron from the liquid side. The formation and distribution of ions in an aqueous solution were investigated. Specifically, the stern layer (SL) is composed of ions strongly adsorbed on the electrode, and the diffusion layer (DL) related to the ion concentration exists at the liquid‐solid interface.^[^ ^37^, ^38^, ^39^ ^]^ Recently, Wang's hybrid EDL model and “Two‐step” formation process have been proposed considering both electron transfer and ions adsorption.^[^ ^37^ ^]^ Herein, we further proposed the multi‐component EDL model to describe the particle behavior at the oil‐solid interface.

**Figure 4:**
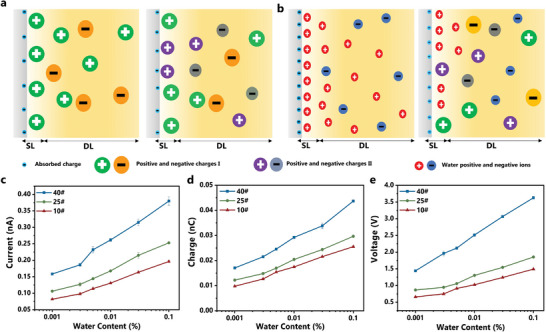
The multi‐component competition mechanism of EDL. a) The composition of oil‐solid EDL with single and multi‐components. b) The composition of oil‐solid EDL with trace water. c–e): The CE performance of transformer oil containing different content of trace water at a steady state. (c) Short‐circuit current, (d) Transferred charge. (e) Open‐circuit voltage.

As shown in **Figure** **4**a, the electron clouds overlap between oil and solid molecules during contact leads to electron transfer at the interface. Herein, oleophobic FEP with strong electron‐capturing performance seizes electrons from oil molecules such as butylbenzene. The electrons from the oil will be trapped in the solid surface state. Subsequently, the free ions will be attracted to the charged interface due to electrostatic interaction. It is worth noting that the ionization reactions generated by ions and electrons are both attributed to the charge transfer and potential distribution. Initially, electron charge transfer plays a dominant role and the solid surface state electrons are removable and relatively unstable compared to the atomic orbital ones.^[^ ^35^ ^]^ Thus, ions interaction through physical/chemical adsorption might change the charge composition of the solid surface to form the final SL. The formation and development of DL are also accompanied by the properties of interface SL charge. Besides, the interface charge kinetics will be changed when the oil contains the second composition. Specifically, the ionization reactions and oil‐solid electron transfer properties differ from the single composition as the two molecules differ in their structural and electronic properties. There exists competition in the composition of solid surface state electrons and ions at this stage, as illustrated in the right part of Figure 4a. The solid interface prefers to interact or adsorb ions with a competitive advantage such as larger charges, which will mainly occupy the surface sites and eventually lead to the formation of EDL with high charge density as well as surface potential.

Further, we considered the EDL development process of oil with trace water to verify the proposed multi‐component competition mechanism. At present, relevant studies on aqueous systems confirm that the L‐S CE output of water is much higher than that of oil.^[^ ^40^, ^41^ ^]^ The reasons include the higher charge carrier content (such as OH, H_3_O^+^), lower viscosity, and the hydrogen interaction in water that contribute to larger surface charge and potential. The EDL structure formed by water and oil with trace water is shown in Figure 4b. The solid side will capture more charges during water‐solid CE owing to the stronger polarity and higher intermolecular hydrogen bonding, bringing larger charge density and higher surface potential. Specifically, the liquid with high polarity generates more charge transfer and shows higher CE output performance.^[^ ^42^ ^]^ And the compositions in transformer oil demonstrate lower polarity than that of water. Besides, the intermolecular hydrogen bonding in water also contributes to superior liquid‐solid CE performance.^[^ ^31^ ^]^ Thus, water has a strong competitive advantage during EDL formation compared to oil. As demonstrated in Figure 4b, the electrons and ion charge contained in the water will occupy or replace surface sites in the EDL, significantly changing the charge density and directly reflecting in the macroscopic electrical signal output characteristics.

As proof of concept, we conducted triboelectric performance tests for three kinds of transformer oils containing trace water. According to the test results shown in Figure 4c–e, the output current of Oil‐Droplet TENG increased significantly with water content. Specifically, the transformer oil with 0.001% (11.17 mg L^−1^) water demonstrated a 26.3%, 18.7%, and 33.4% increase in short‐circuit current, and transferred charge and open‐circuit voltage. When the trace water content reached 0.01%, the triboelectric output increased by 100%–125%. Relevant results confirmed that the trace water could significantly affect the oil‐solid triboelectrification properties, verifying the rationality of the proposed multi‐component EDL model and the advanced charge competition performance of water. Besides, it provides a theoretical basis for real‐time online detection of trace water in transformer oil by oil‐solid TENG.

### 2.5 Tubular‐TENG for Self‐Powered Trace Water Detection in Transformer Oil

**Figure 5:**
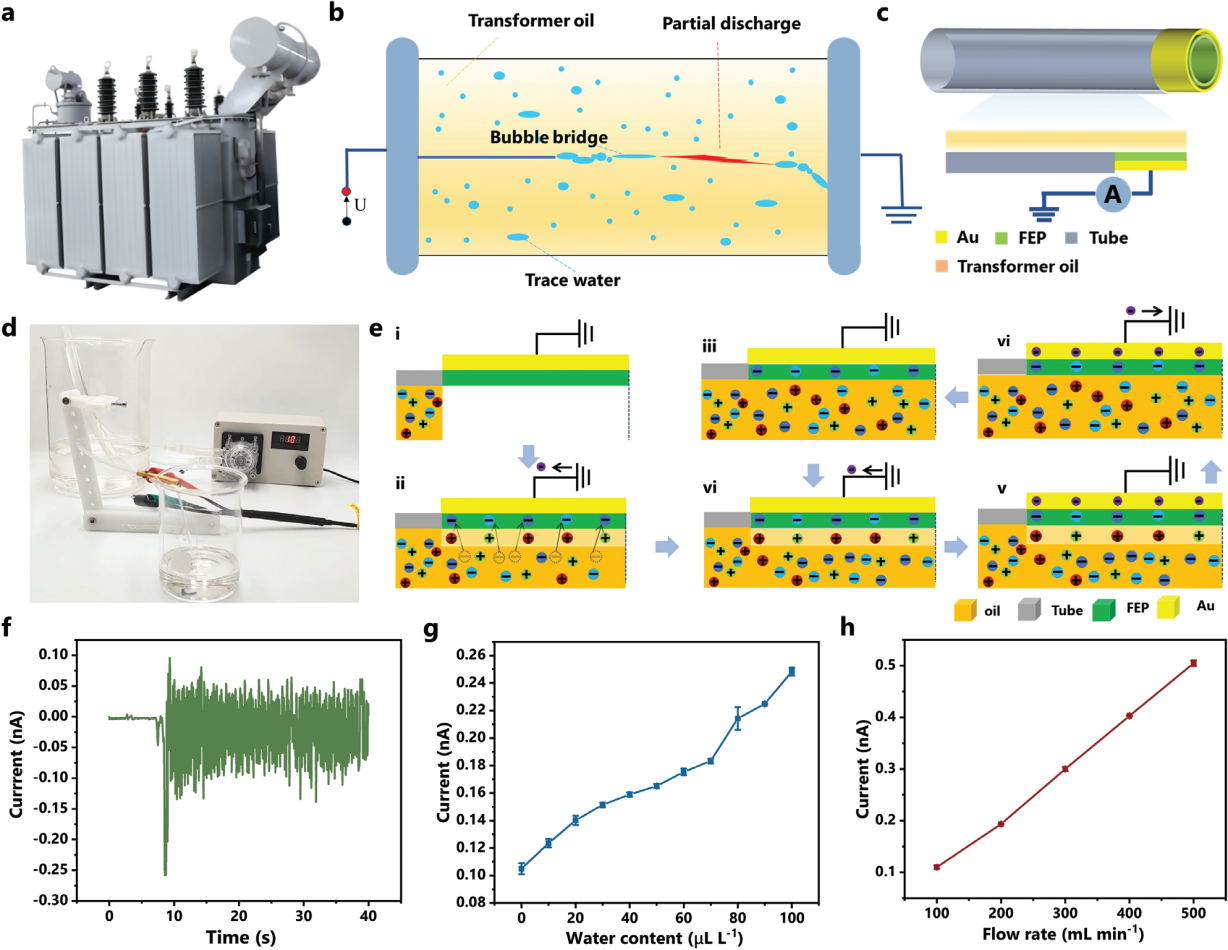
Tubular‐TENG as a self‐powered sensor for trace water detection in transformer oil. a) Schematic diagram of the oil‐immersed transformer. b) The formation of water‐induced “bubble bridge” in transformer oil. c) The structure of Tubular‐TENG. d) The composition of oil flowing platform with Tubular‐TENG for self‐powered trace water detection. e) The working principle of the Tubular‐TENG. f) The output waveform of the Tubular‐TENG. g) The short‐circuit current response of insulating oil containing different trace water content. h) The influence of oil flowing speed on output characteristics of Tubular‐TENG.

Oil‐immersed transformer plays a significant core role in energy conversion and transmission in the power grid to step‐up or step‐down the line voltage. The reliability of oil‐paper insulation and equipment operation highly relies on the transformer oil quality, which undertakes the electrical insulation and heat dissipation. Commonly, the 25^#^ transformer oil is mainly used and the fundamental physical, chemical parameters, and quality strictly follow relevant IEEE or GB standards, as listed in Table S1, Supporting Information.^[^ ^43^, ^44^, ^45^ ^]^ As illustrated in **Figure** **5**a,b, trace water in transformer oil is one of the most critical factors that endanger transformer insulation. The presence of water could lead to an acceleration of transformer oil aging and a reduction of oil insulation performance owing to the formation of bubbles after heating. The “bubble bridge” in transformer oil will lead to partial discharge and even breakdown of transformer oil, endangering the equipment's safe and stable operation. Normally, transformer oil flows during actual operation due to force or temperature difference‐induced circulation. This brings the feasibility of utilizing the oil‐solid TENG for oil flow energy harvesting in transformers and self‐powered composition sensing without an external power supply.

According to the multi‐component EDL mechanism explored above, we proposed a Tubular‐TENG as a self‐powered sensor for trace water detection in transformer oil. The overall structure of the device is shown in Figure 5c. The oleophobic FEP film was used as the trace water detection layer with the gold‐sputtered on its reverse side as an electrode for charge induction. The nylon tube acted as the support layer for the device. The structure of the transformer oil flow simulation system was given in Figure 5d, the Tubular‐TENG as the detection probe was connected to the oil pipeline with a peristaltic pump for flowing speed control. The Tubular‐TENG output was collected and imported into the data acquisition system. The working principle of the self‐powered sensor is shown in Figure 5e. Briefly, oil‐solid CE first generates electron transfer at the interface, accompanied by the ionization reaction of oil and water molecules. The oleophobic FEP film was negatively charged with surface potential built at this stage (Figure 5e(i,ii)). Then the EDL is built with ions, electrons adsorbed on the oleophobic FEP as the stern layer and the free ions or electrons are attracted to the electrified surface owing to electrostatic interaction as the diffusion layer. However, the oil flow changes the dynamic equilibrium of the formed EDL. The surface‐induced charge can be measured during the reconstruction and destruction of EDL (Figure 5e(iii–vi)).

Figure 5f and Movie S3, Supporting Information, demonstrate the output waveform of the Tubular‐TENG. There existed a transient process when the peristaltic pump was started to drive the oil flowing through the pipeline. Then the high‐peak value at the initial time rapidly decayed to a stable value, which was the same as the current variation of the Oil‐Droplet TENG described above. Oil flow can be regarded as highly high‐frequency repetition and superposition of oil droplets. The output waveforms are correlated as a result of their similar EDL formation and development processes. The pulse signals also verified the proposed working principle in Figure 5e. Moreover, the pulse waveforms overlapped and led to continuous waveforms (Figure 5f) owing to a large number of droplets contained in the oil flow. The Tubular‐TENG overcomes the defect of the non‐obvious negative polarity output of the Oil‐Droplet TENG because of the high viscosity of the oil. Its output signal is a bipolar waveform with a positive peak value higher than a negative peak value, and the imbalance between positive and negative peak values is part of the charge carried away by the oil flow, which is consistent with the previous results.

The triboelectric output performance of the 25^#^ transformer oil with 10–100 µL L^−1^ trace water at a flow rate of 100 mL min^−1^ tested by the Tubular‐TENG is shown in Figure 5g. It can be found that the output current increased significantly with the water content in the oil. The oil containing 10 µL L^−1^ water demonstrated an output of 0.135 nA, which was 35% higher than pure insulating oil. It proved that the Tubular‐TENG could be applied in self‐powered trace water detection in transformer oil. Further, the influence of the oil flow rate on the output was investigated. As shown in Figure 5h, the device's response demonstrated an almost linear increase trend as the oil flow gradually increased from 100 to 500 mL min^−1^ and the sensitivity of the Tubular‐TENG reached 1.5 pA µL L^−1^. This indicated that the simple calibration of the device could meet various operating conditions of the transformer. The fabricated Tubular‐TENG is a susceptible self‐powered sensor with promising application in real‐time online trace water detection in transformers.

## 3 Conclusion

In summary, we explored the oil‐solid CE mechanism based on the Oil‐Droplet TENG as a probe to investigate the charge transfer and surface potential development process. The electron and ion charge transfer processes existed, and the former initially dominated the oil‐solid triboelectrification. The oil molecular with higher HOMO energy level and concentrated electron function wave function distribution endows higher oil‐solid triboelectric output performance. A competitive effect exists in the multi‐component oil system, which affects the EDL formation process by binding with surface sites. As a proof of concept, the Tubular‐TENG based self‐powered trace water sensor for the transformer was fabricated, demonstrating a relatively high detection limit of 10 µL L^−1^ and a response range of 10–100 µL L^−1^. This work clarified the oil‐solid CE mechanism and charge transfer competition mechanism in EDL. Meanwhile, it provides a new route for trace water detection in oil for the next information perception and intelligent operation of transformers in the power industry.

## 4 Experimental Section

### Materials

1H,1H,2H,2H‐Perfluorodecyltriethoxysilane or POTS were purchased from Deep Blue Reagent Co., Ltd. FEP film was purchased from Fluorine Plastic Materials Co., Ltd. The 10^#^, 25^#^, and 45^#^ transformer oils were obtained from MOROKE Co., Ltd. The N‐dodecane (>98%), n‐hexadecane (>98%), cyclooctane (>98%), and n‐butylbenzene (>99%) were purchased from Shanghai Aladdin Biochemical Technology Co., Ltd.

### Fabrication of TENGs

The TENG includes Oil‐Droplet TENG for oil‐solid CE characterization and Tubular‐TENG for self‐powered trace water detection in transformer oil. Fabrication of Oil‐Droplet TENG: The FEP film (15 cm *5 cm * 0.05 mm) was oleophobic treated by drop coating POTS on its surface. Specifically, the FEP film was first treated with alkaline (7.5 m NaOH) for 3 h at 70 °C, followed by DI water washing and drying at 70  °C for 12  h. Then FOTS was drop coated on the hydroxylate FEP film and left at ambient conditions (25 °C) for 24 h until curing. The silanization coupling reaction occurs between the ‐OH and silane of POTS to form chemical bonds. Subsequently, a layer of gold (5 cm * 5 cm) was deposited on the middle region of the FEP film reverse surface as the electrode. The gold sputtering was conducted at 50 mA for 90–120 s (layer thickness of 22.5–30 nm). Then the oleophobic FEP film with an electrode attached was glued to the PMMA substrate. Before the oil‐solid CE test, the plasma ion air gun was employed to blow the oleophobic FEP surface to remove the residual charge on the film. Tubular‐TENG: The nylon tube (20 cm length, 4 mm * 6 mm) was selected as the transformer oil channel. The oleophobic FEP film with the gold electrode (2.5 cm length, 4 mm diameter) was adhered to the inner wall of the nylon tube to obtain the Tubular‐TENG. The other side of the nylon tube was connected to the oil outlet of the peristaltic pump.

### Characterization and Measurement

The surface morphology and element distribution of oleophobic FEP film were characterized by a field emission electron microscope with EDS (EPMA; JXA‐8530F Plus; JEOL Co. ltd, Japan). The static oil contact angle was measured by Dataphysics OCA15 Pro with a droplet of 5 µL. As for the oil‐solid CE tests based on Oil‐Droplet TENG, the microflow pump (SPL Lab01, Easyjump Co., Ltd.) connected with a glass syringe was used to squeeze oil droplets, and the needle was connected with grounded copper wire to remove the charges in the droplets. The oleophobic FEP film was adjusted to a suitable angle and position to avoid direct contact with oil droplets on the electrode area. The oil with extra content trace water was prepared by adding corresponding liquid water into the oil and then magnetic stirring for 2 h. Then the sample was calibrated using the moisture titration device (JZWS‐9000A, JAN ZONG Co., Ltd.) based on the Karl Fischer Coulometric titration method. The trace water content interval was set as 0.001%–0.1% (11.17–1117 mg L^−1^), which covered the transformer oil water content (15–35 mg L^−1^) stipulated by the national standard GB/T7595‐2008 entitled “Quality of transformer oil in service”. The electrometer (6517B, Keithley) connected with the multimeter (DAQ 6510, Keithley) was employed to measure the short‐circuit current, open‐circuit voltage, accumulated/transferred charge, and a personal computer with TPS‐4000 software recorded the acquired data. The peristaltic pump (PC03) with the adjustable flow range of 0.15–610 mL min^−1^ and the control accuracy of 1mL min^−1^ was used to provide transformer oil flow.

### Calculation Method

The oil‐solid CE development process was simulated based on the AC/DC module of COMSOL Multiphysics. All the spin unrestricted DFT calculations were performed using the Dmol^3^ package of Materials Studio.^[^ ^46^ ^]^ Specifically, the double numerical plus polarization (DNP) was selected as the atomic orbital basis set.^[^ ^47^ ^]^ The B3LYP method was employed to describe the electron exchange and correlation.^[^ ^48^ ^]^ The global orbital cut‐off radius was set to 4.0 Å. The convergence criteria for geometric optimization were set as follows: 1) 1.0 e^−5^ Hartree (Ha) on energy, 0.002 Ha Å^−1^ on Max. force and 0.005 Å on Max. displacement. The frequency analysis was also conducted to confirm the stability of molecules.

### Statistical Analysis

- Pre‐processing of data: basal Butterworth low‐pass filter was used to filter high‐frequency environmental interference signals (mainly 50 Hz). The filter cut‐off frequency was set at 3 Hz, the maximum passband attenuation was 1 dB, and the stopband attenuation was 60 dB.
- Data presentation: in Figures 4c–e and 5g,h, the short‐circuit current was presented with the mean plus and the error bar.
- Sample size (n) for each statistical analysis: in Figures 4c–e and 5g,h, data were taken from the average of 20 data points of steady‐state peak current, and data points in the figure were taken from the average of four average current points.
- Statistical methods used to assess significant differences with sufficient details: uninvolved.
- Software used for statistical analysis: MATLAB 2017b.

## Conflict of Interest

The authors declare no conflict of interest.

## Supporting information

Supporting Information

Supporting Movie 1

Supporting Movie 2

Supporting Movie 3

## Data Availability

The data that support the findings of this study are available from the corresponding author upon reasonable request.
