# Supplementary material for: Triboelectric Mechanism of Oil‐Solid Interface Adopted for Self‐Powered Insulating Oil Condition Monitoring
Source: Adv Sci (Weinh). 2023 Feb 24;10(13):2207230. doi: 10.1002/advs.202207230 (PMC10161025; doi:10.1002/advs.202207230)
Supplement: Supplementary file 1 — Supporting Information [file ADVS-10-2207230-s004.pdf]

ADVANCED SCIENCE
Open Access

Supporting Information

for *Adv. Sci.*, DOI 10.1002/advs.202207230

Triboelectric Mechanism of Oil-Solid Interface Adopted for Self-Powered Insulating Oil Condition Monitoring

*Song Xiao, Haoying Wu, Nan Li, Xiangyu Tan, Haocheng Deng, Xiaoxing Zhang, Ju Tang and Yi Li**

Supporting Information

Triboelectric Mechanism of Oil-solid Interface Adopted for Self-powered Insulating Oil Condition Monitoring

*Song Xiao#, Haoying Wu#, Nan Li, Xiangyu Tan, Haocheng Deng, Xiaoxing Zhang, Ju Tang, Yi Li***

[#]The authors contribute equally

Dr. S. Xiao, Mr. H. Y. Wu, Dr. H. C. Deng, Prof. J. Tang, Prof. Yi Li

[*]Corresponding author Email: li_yi@whu.edu.cn

School of Electrical Engineering and Automation, Wuhan University, Wuhan, Hubei 430072, China.

Dr. N. Li

State Grid Tianjin Electric Power Research Institute, Tianjin 300392, China

Dr. X. Y. Tan

Electric Power Research Institute, Yunnan Power Grid Co., Ltd., Kunming, Yunnan 650217, China.

Prof. X. X. Zhang

Hubei Engineering Research Center for Safety Monitoring of New Energy and Power Grid Equipment, Hubei University of Technology, Wuhan, Hubei 430068, China.

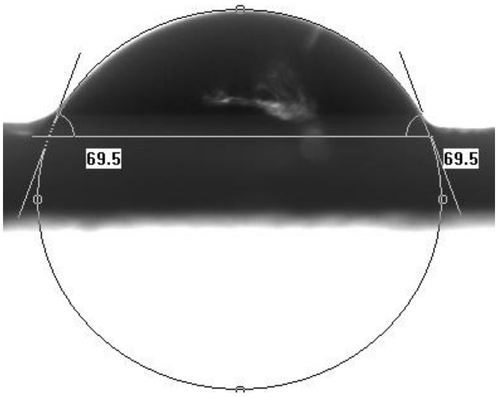

Figure S1. The oil contact angle of $25^{\#}$ transformer oil with untreated commercial FEP.

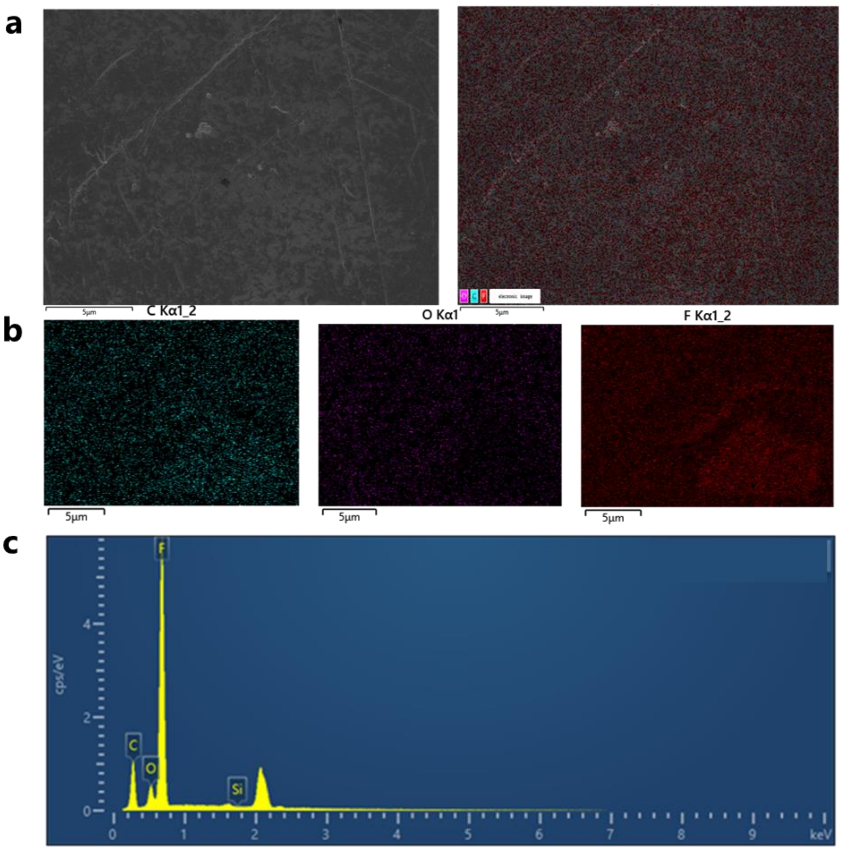

Figure S2. SEM-DES of the treated oleophobic FEP film. (a) Morphology of the oleophobic FEP. (b) Element distribution of C, F and O. (c) Spectrum of the EDS distribution map.

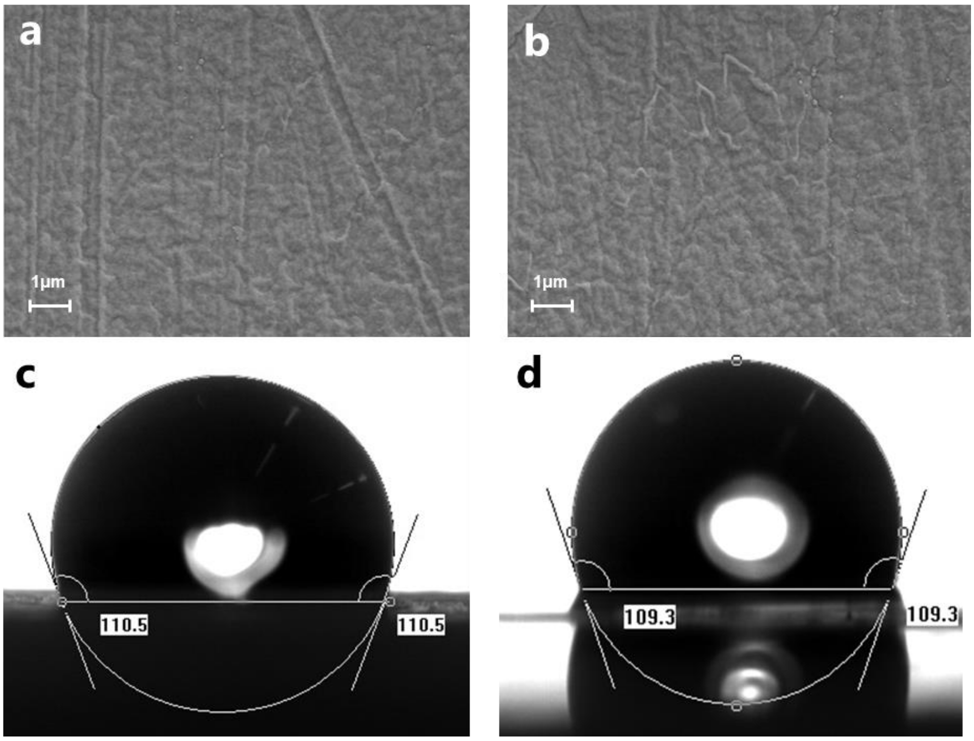

Figure S3. The SEM and oil contact angle of oleophobic FEP film before and after long-term (6h) oil-solid CE tests. a) SEM before tests. b) SEM after tests. c) Oil contact angle before tests. d) Oil contact angle after tests.

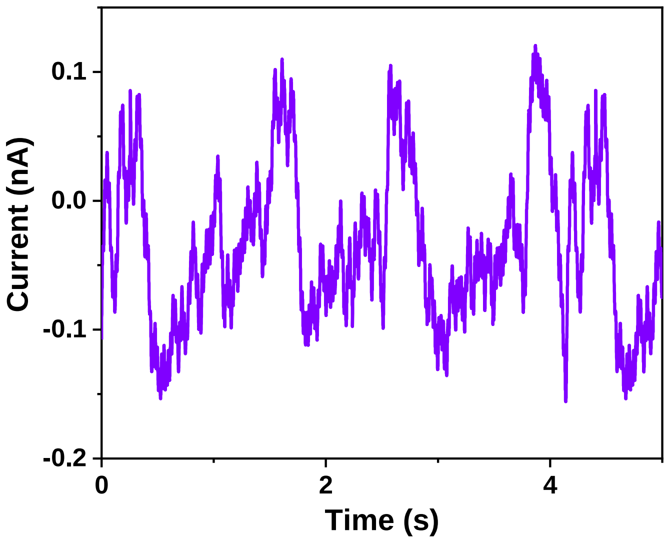

Figure S4. The induced current signal of untreated commercial FEP film

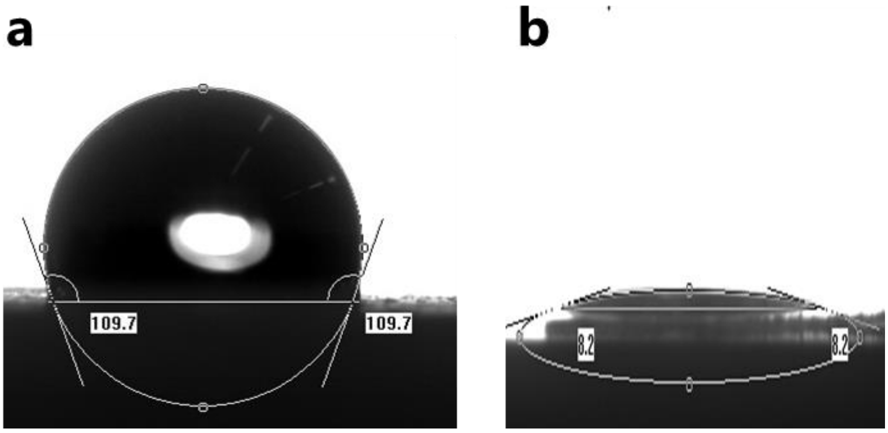

Figure S5. The oil contact angle of the solid film after a set of CE tests (120 s, based on 25$^{\#}$ transformer oil). a) Oleophobic FEP. (b) untreated commercial FEP film.

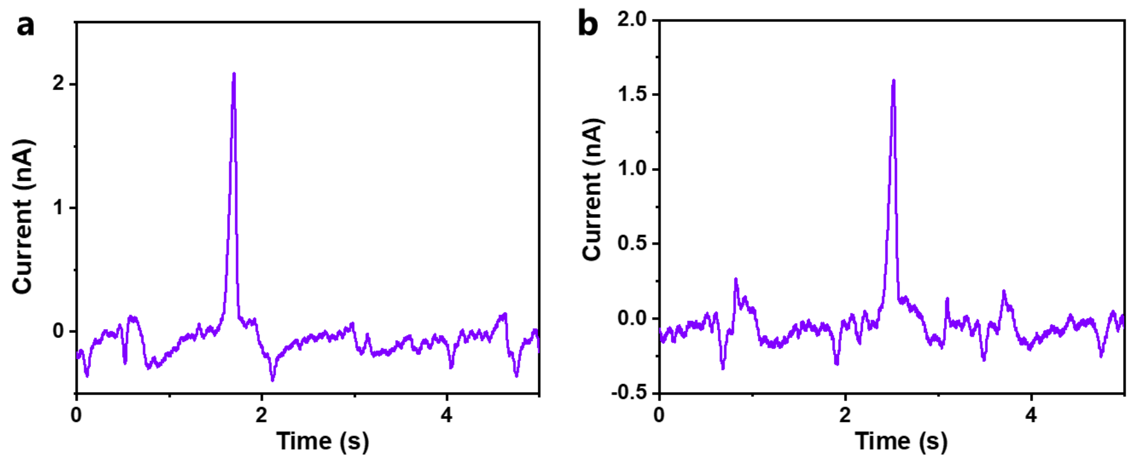

Figure S6. The induced current signals by oil droplets falling on the oleophobic FEP film. (a) The first oil droplet. (b) The 40th oil droplet.

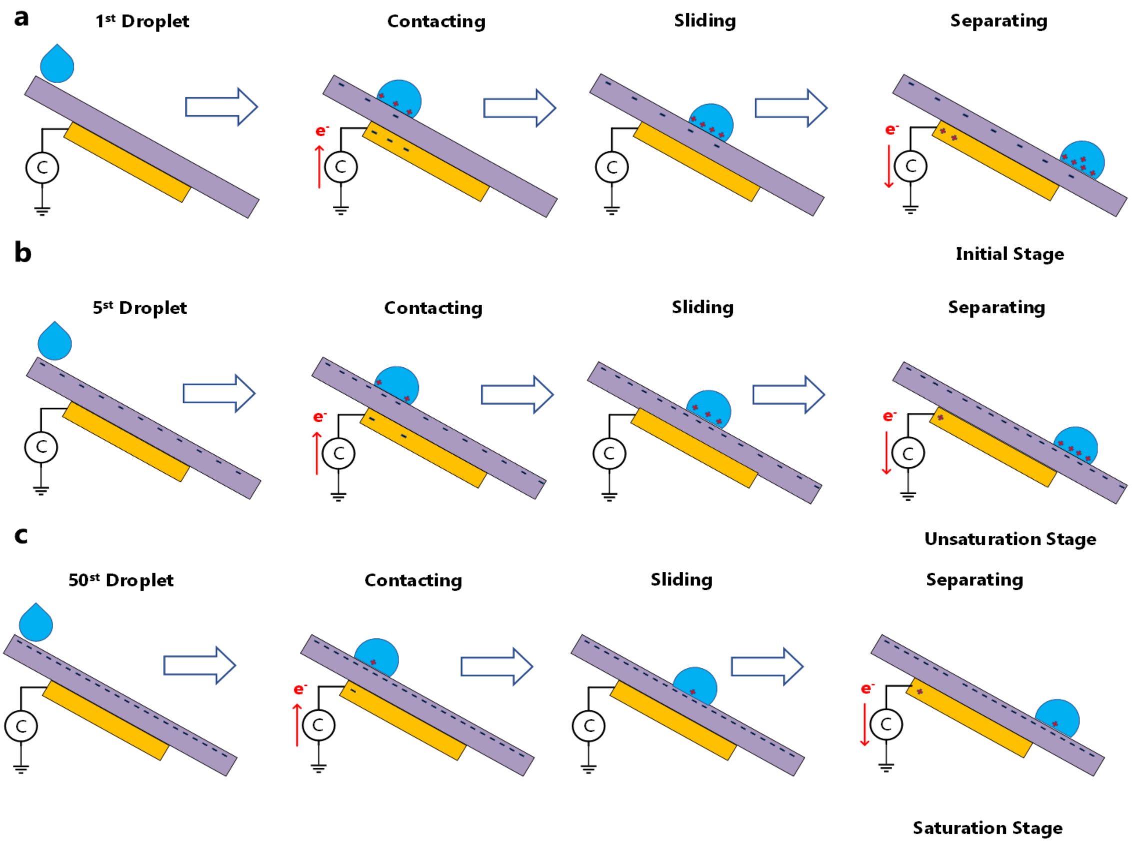

Figure S7. Charge transfer process of the Oil-Droplet TENG. Three stages including initial, unsaturation, and saturation stages are included. (a) Initial stage: For the first oil droplet, the negative charge on the oleophobic FEP film is zero, and the charge induced by the oil droplet is the largest. (b) Unsaturation stage: In the unsaturated stage, surface charge of the oleophobic FEP film increases with the oil droplets number, and the induced charge gradually decreases. (c) Saturation stage: As the charge carried by the oleophobic FEP film is saturated, the transferred and induced charge remains stable during the oil droplet sliding process.

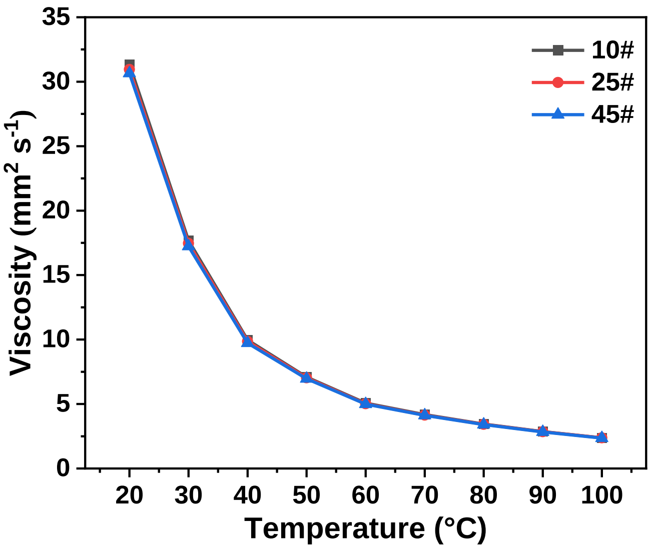

Figure S8. The viscosity of three types of transformer oils (10#, 25# and 45#).

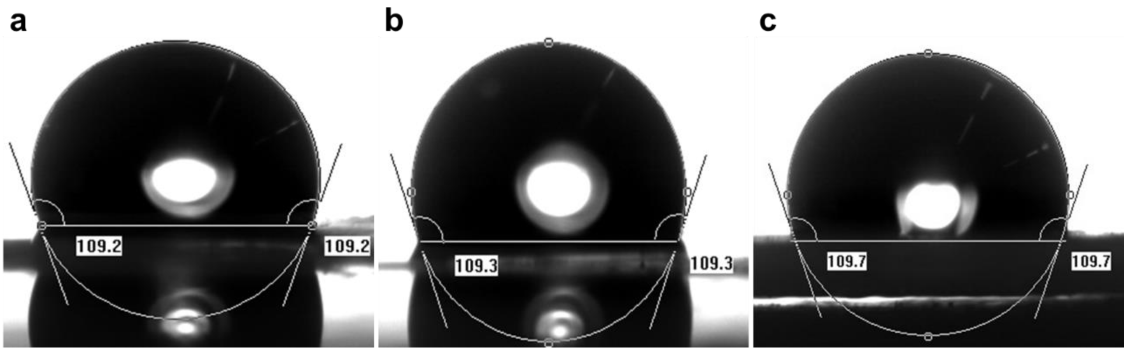

Figure S9. The oil contact angle of three types of transformer oils. a) 10# b) 25# c) 45#.

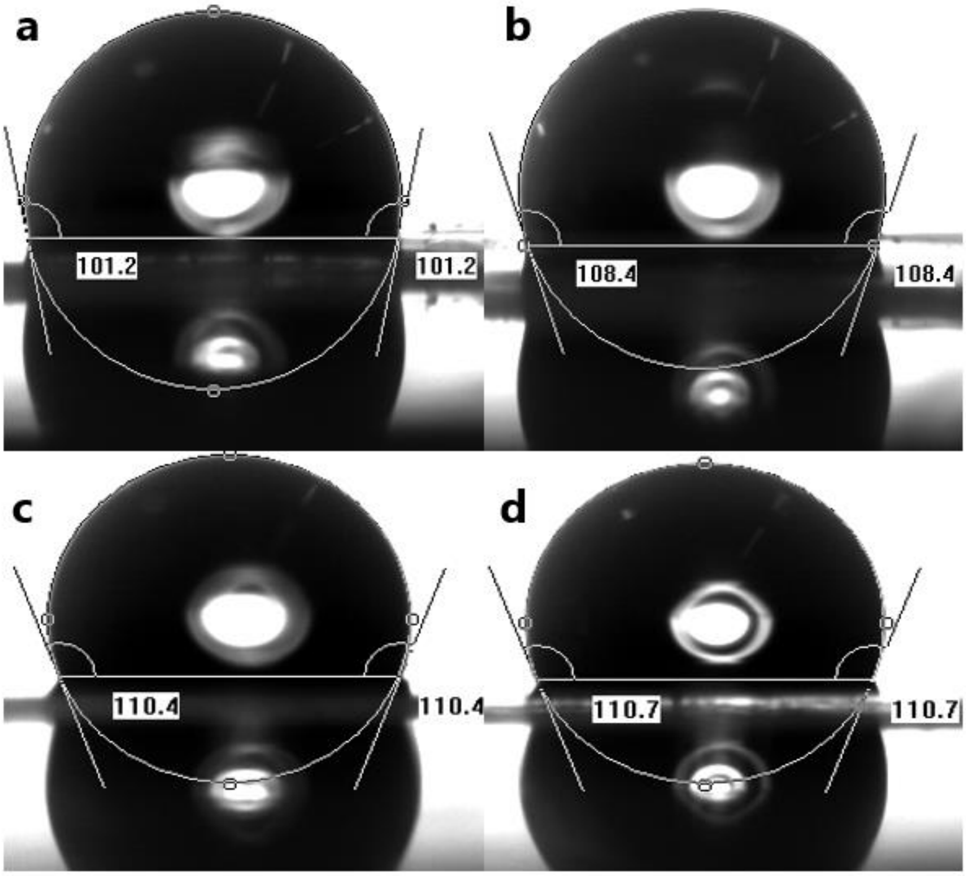

Figure S10. The oil contact angle of four types of oils. a) n-dodecanoic b) n-hexadecane c) cyclooctane and d) butylbenzene.

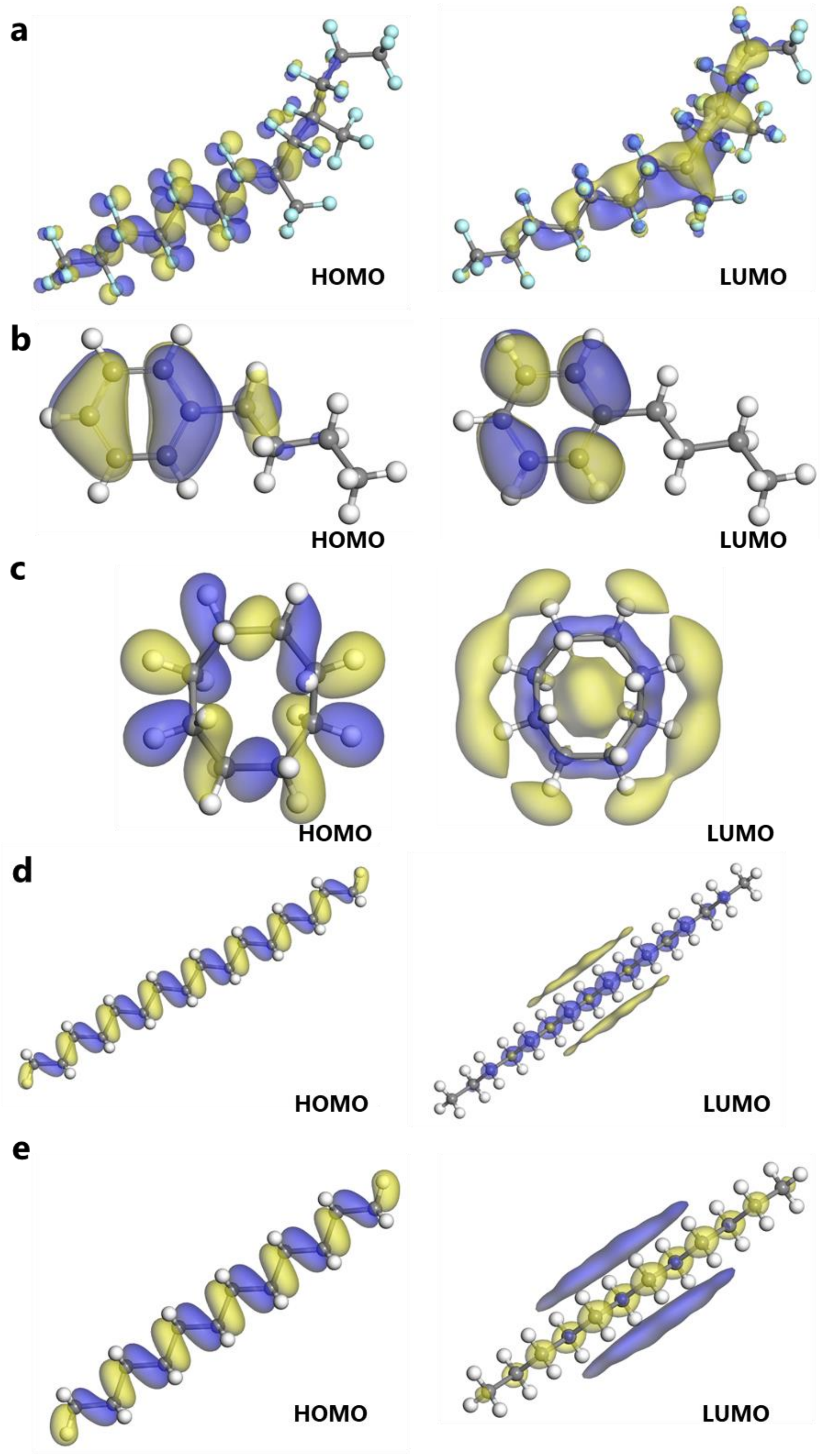

Figure S11. The HOMO and LUMO orbital wave function distribution of FEP and transformer oil compositions. (a) FEP. (b) butyl benzene. (c) cyclooctane. (d) n-hexadecane. (e) n-dodecane.

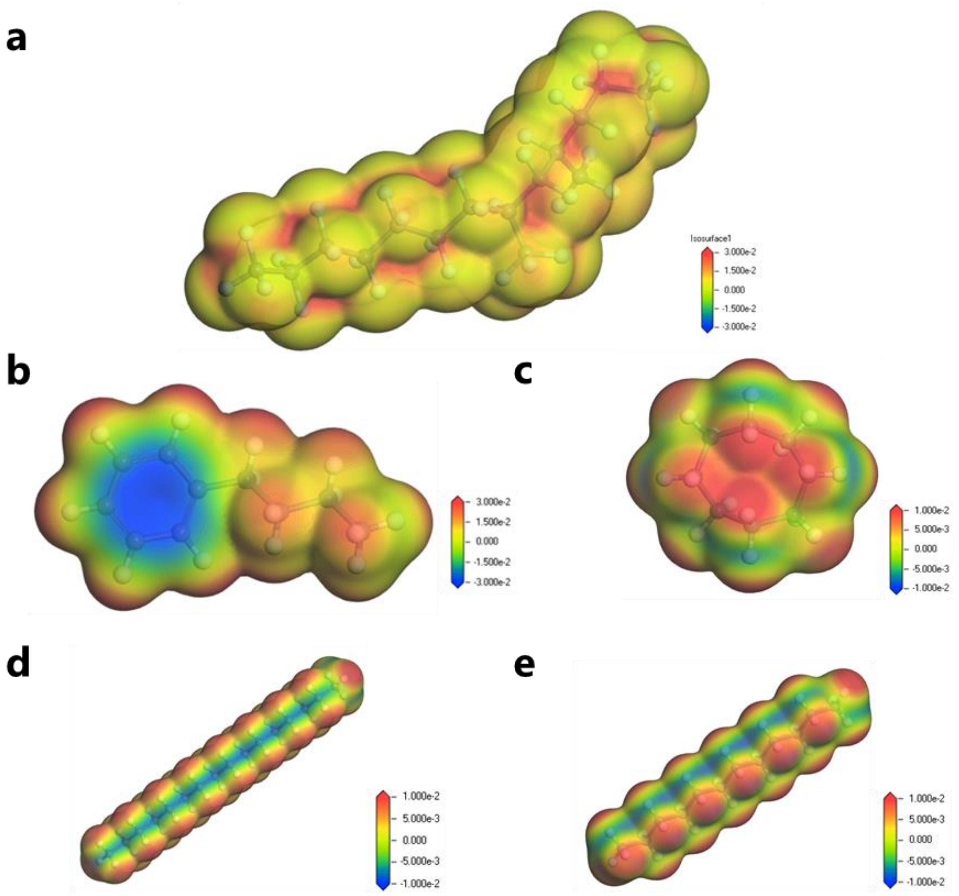

Figure S12. The electrostatic potential distribution of FEP and transformer oil compositions. (a) FEP. (b) butyl benzene. (c) cyclooctane. (d) n-hexadecane. (e) n-dodecane.

Table S1 The basic quality parameters of transformer oils[1].

No	Item	Voltage level (kV)	Quality index	
			New oil	Operation oil
1	Water-soluble acid (pH)	/	＞5.4	≥4.2
2	Acid value (mg KOH g^{-1})	/	≤0.03	≤0.1
3	Flash point (℃)	/	≥135	
4	Trace water content (mg L^{-1})	330-1000	≤10	≤15
		220	≤15	≤25
		≤ 110	≤20	≤35
5	Surface tension (mM m^{-1})	/	≥35	≥19
6	Dielectric loss factor (90℃)	500-1000	≤0.005	≤0.020
		≤330	≤0.010	≤0.040
7	Dielectric strength (kV)	750-1000	≥70	≥60
		500	≥ 60	≥ 50
		330	≥ 50	≥ 45
		66-220	≥ 40	≥ 35
		≤ 35	≥ 35	≥ 30
8	Volume resistivity (Ω m)	500-1000	$\geq 6*10^{10}$	$\geq 1*10^{10}$
		≤330	$\geq 6*10^{10}$	$\geq 5*10^{9}$

References

[1] GB/T7595-2008, Quality of transformer oil in service, 1-10, 2009.
